# Hospital Re-Admission Prediction Using Named Entity Recognition and Explainable Machine Learning

**DOI:** 10.3390/diagnostics14192151

**Published:** 2024-09-27

**Authors:** Safaa Dafrallah, Moulay A. Akhloufi

**Affiliations:** Perception, Robotics and Intelligent Machines (PRIME), Department of Computer Science, Université de Moncton, Moncton, NB E1A 3E9, Canada; safaa.dafrallah@umoncton.ca

**Keywords:** hospital readmission, MIMIC-III, named entity recognition, clinical notes, discharge summaries, machine learning, explainable AI

## Abstract

Early hospital readmission refers to unplanned emergency admission of patients within 30 days of discharge. Predicting early readmission risk before discharge can help to reduce the cost of readmissions for hospitals and decrease the death rate for Intensive Care Unit patients. In this paper, we propose a novel approach for prediction of unplanned hospital readmissions using discharge notes from the MIMIC-III database. This approach is based on first extracting relevant information from clinical reports using a pretrained Named Entity Recognition model called BioMedical-NER, which is built on Bidirectional Encoder Representations from Transformers architecture, with the extracted features then used to train machine learning models to predict unplanned readmissions. Our proposed approach achieves better results on clinical reports compared to the state-of-the-art methods, with an average precision of 88.4% achieved by the Gradient Boosting algorithm. In addition, explainable Artificial Intelligence techniques are applied to provide deeper comprehension of the predictive results.

## 1. Introduction

Early hospital readmissions have a very negative impact on both healthcare systems and patients. According to Krumholz et al. [1], readmitted patients have a mortality rate of 24.56%, compared to only 11.17% for non-readmitted patients. A high readmission rate financially burdens the healthcare system; as reported by the Agency for Healthcare Research and Quality (AHQR), the average cost of readmitted patients in the USA was approximately USD 15,000 in 2018 [2].

To tackle this issue, several studies have been conducted to understand the factors behind high readmission rates and to propose predictive models. A number of relevant studies relate to predicting unplanned hospital readmissions using discharge summaries.

Liu et al. [3] proposed a deep learning approach for predicting heart failure readmission from clinical notes using unstructured clinical notes from the MIMIC-III database. They utilized both CNN and traditional machine learning models, initially representing the clinical notes as sequences of word embedding vectors obtained through the word2vec technique and pretraining the models on PubMed text articles and abstracts. These embedding vectors served as inputs for training the CNN model. For interpretability, chi-square scoring was used on correctly predicted samples to identify the key features related to readmission. Their approach achieved F1-scores of 75.6% for general readmission and 73.3% for 30-day readmission prediction, compared to 67.4% and 65.6%, respectively, for the random forest model.

In a related study, Lineback et al. [4] predicted 30-day readmission after stroke from Electronic Health Records (EHRs) using machine learning and natural language processing methods. The proposed approach combined discrete variables (demographics, comorbidities, number of hospitalizations, etc.) and non-discrete variables from unstructured text-based clinical notes, including admission, progress, consultation, and discharge notes. To train the models, the authors constructed four different features: discrete features, bag of words, principal component analysis (PCA), and word vectors. In addition, they proposed two prediction methods: the ensemble feature model, which trains different machine learning models on a combination of all features and then uses a meta-classifier for final prediction, and the classifier ensemble model, which trains individual models on separate features and then combines their results for meta-classification. The ensemble classifier model achieved an Area Under the Curve (AUC) of 65% for all-cause readmission and 64% for stroke readmission, compared to 64% and 63% for the feature ensemble method.

Wu et al. [5] proposed deep learning models for Named Entity Recognition (NER) from clinical texts, employing Convolutional Neural Network (CNN) and Recurrent Neural Network (RNN) architectures.

Rumshisky et al. [6] used natural language processing on narrative discharge summaries to predict early hospital psychiatric readmission. The authors trained a 75-topic Latent Dirichlet Allocation (LDA) model to identify groups of words associated with psychiatric symptoms and major depressive disorder comorbidities. A Support Vector Machine (SVM) model was used as a baseline. The experimental results were based on inpatient discharge narrative notes of 4687 patients with major depression, of whom 470 were readmitted within 30 days. Using LDA topics improved readmission prediction, with an Area Under the Receiver Operating Characteristic Curve (AUC-ROC) of 0.784 compared to 0.618 for the baseline.

Golmaei et al. [7] proposed a hybrid deep learning model called DeepNote-GNN for predicting hospital readmissions using clinical notes and patient networks. The proposed framework extracts deep representations from clinical notes using a pretrained BERT model, then builds a patient network from these representations using a Graph Neural Network (GNN) model. According to the authors, the proposed DeepNote-GNN framework achieved a higher performance compared to state-of-the-art baselines on MIMIC-III dataset, with an AUC-ROC of 0.858 compared to 0.768 using the ClinicalBert model [8].

Orangi-Fard et al. [9] proposed an Intensive Care Unit (ICU) readmission prediction using bag-of-words approach and support vector machines with the radial basis function kernel (SVM-RBF). They constructed a document–term matrix with 3000 features from discharge summaries, performed feature selection to identify 825 dominant features, and then trained machine learning models. According to the authors, the feature selection technique achieved an AUC-ROC of 0.74 compared to 0.71 without feature selection.

The main purpose of the current paper is to predict unplanned hospital readmissions from discharge summaries using NLP and machine learning techniques. Therefore, our first aim is to understand the factors leading to readmission. On this basis, we propose a predictive study that can be deployed inside a decision support tool, allowing caregivers to identify high-risk patients. Unlike our previous work on readmission prediction using Electronic Health Record (EHR) data from the MIMIC-III database [10], this study focus on using discharge notes, as they may contain additional information that is not provided by EHRs.

To this end, we conducted a descriptive analysis study in which we extracted the most frequent diagnoses for all admission types as well as those diagnoses with the highest readmission rate and highest death rate. Then, we extracted relevant features from the preprocessed dataset using NLP and NER techniques. In order to restrain the length of the extracted features, we focused on only the 100 most frequent words related to the diagnoses with the highest readmission rate, which included the name of diseases, symptoms, and chemicals. These extracted entities were later used as input features to machine learning models for readmission prediction. Finally, explainable AI approaches were applied to highlight the features with the highest impact on the decision process.

The rest of this paper is organized as follows: Section 2 presents a descriptive analysis for MIMIC-III clinical database, followed by a detailed explanation of the preprocessing steps; Section 3 presents the techniques used to extract entities from discharge summaries, which are thereafter used as input features to ML models; explainable AI is used to in Section 4 to interpret the results of the best predictive model and select the features with the highest importance; finally, we discuss the results and conclude the work in Section 5 and Section 6, respectively.

## 2. Methods

### 2.1. Dataset

MIMIC-III [11] is a large and freely available clinical database comprising Electronic Health Record (EHR) data gathered between 2001 and 2012 from patients admitted to the Intensive Care Unit (ICU) of Beth Israel Hospital. The database consists of 26 tables, including ‘ADMISSIONS’, ‘PATIENTS’, ‘NOTEEVENTS’, ‘DIAGNOSES_ICD’, and ‘D_ICD_DIAGNOSES’. The main important table is ‘ADMISSIONS’, which contains 58,976 distinct admissions belonging to 46,520 patients. Each admission and each patient has a unique identifier. In addition to the unique identifiers for each admission and patient, each admission record contains information concerning the admission type (elective, emergency, urgent, newborn), time of admission, time of discharge, diagnosis at time of admission, death time, etc. The ‘PATIENTS’ table provides more information concerning patients’ gender and age. Along with ‘ADMISSIONS’ table, the ‘NOTEEVENTS’ table is the most important for our work, as it contains significant clinical notes for each admission related to discharge summaries, ECG reports, radiology reports, etc.

**Figure 1:**
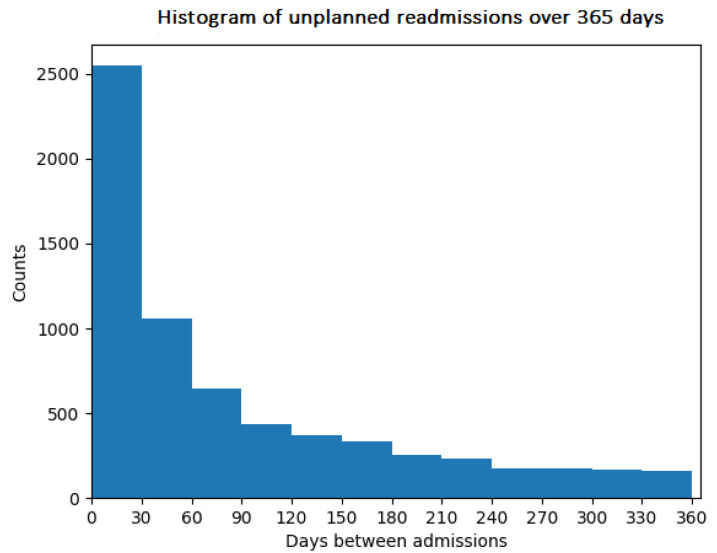
Histogram of unplanned readmissions over 365 days.

In this work, we are mostly interested in data related to the type of admission, time of admission, and time of discharge, which allow the number of days between discharge and the next unplanned admission to be computed, as well as data on the diagnosis and discharge summaries, which are used by the predictive model. As we are only interested in unplanned readmissions occurring within 30 days of discharge, we create output labels, where a positive label represents a readmission occurring within 30 days of discharge and a negative label represents any other situation. Figure 1 shows a histogram plot of the number of readmissions over 365 days; it can be seen that readmissions within 30 days are the most frequent, representing 38.76% of all readmissions within the year, for a total of 2549 cases.

### 2.2. Descriptive Analysis

**Figure 2:**
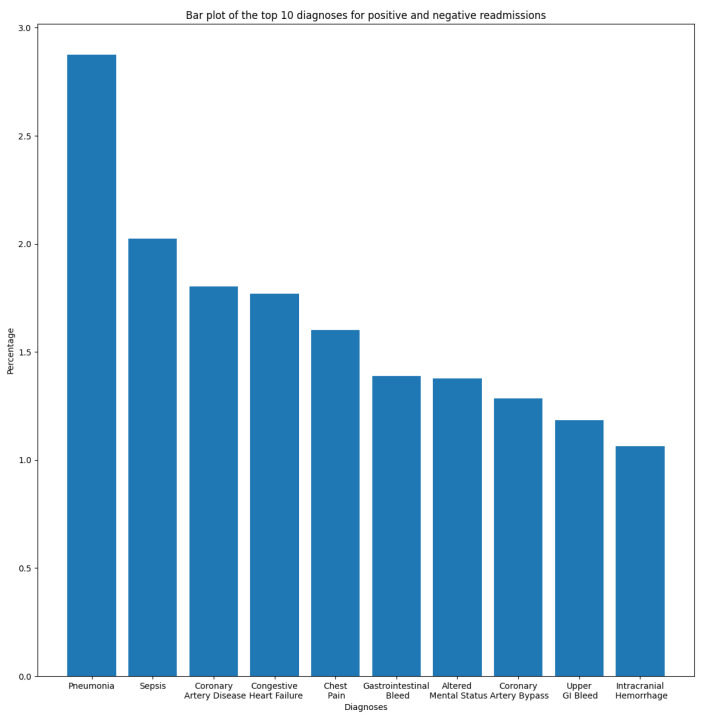
Top ten diagnoses for all admissions.

**Figure 3:**
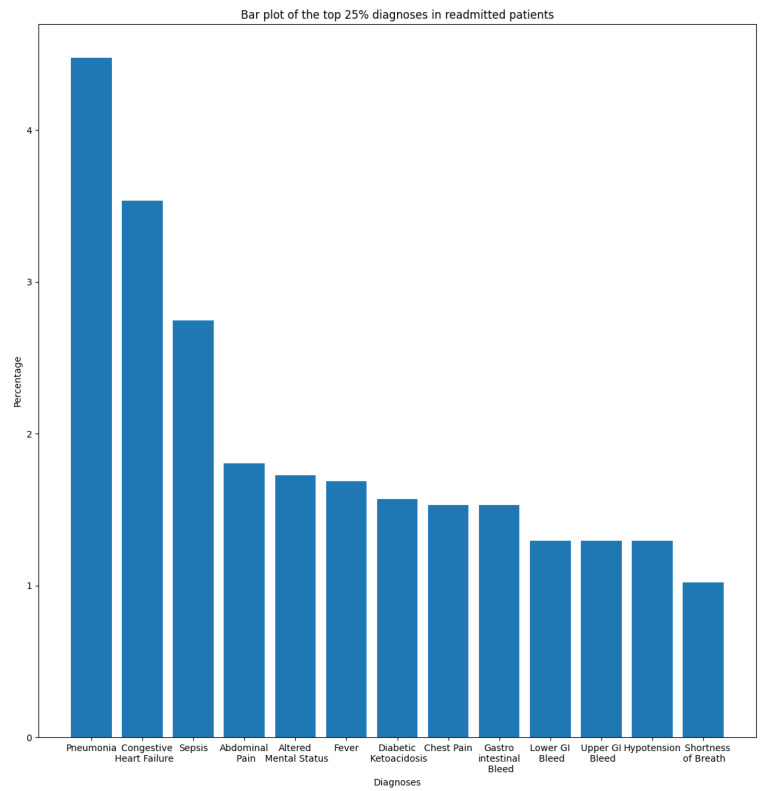
Top 25% primary diagnoses of readmitted patients.

**Figure 4:**
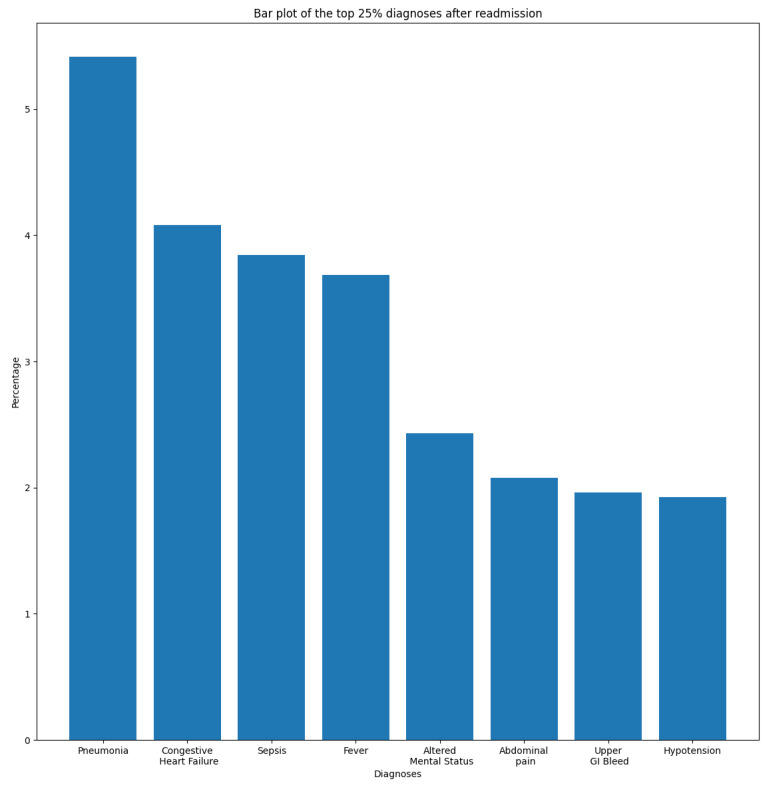
Top 25% of post-readmission diagnoses.

**Figure 5:**
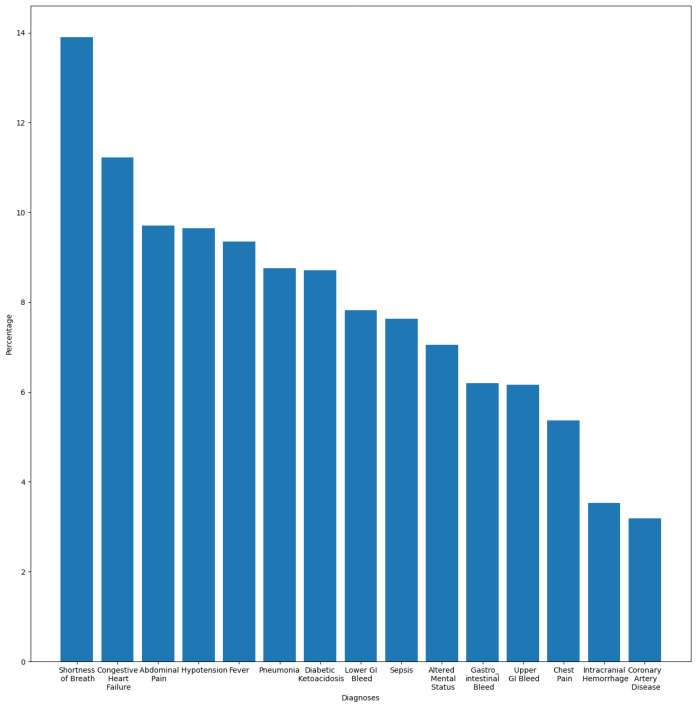
Diagnoses with the highest readmission rate.

**Figure 6:**
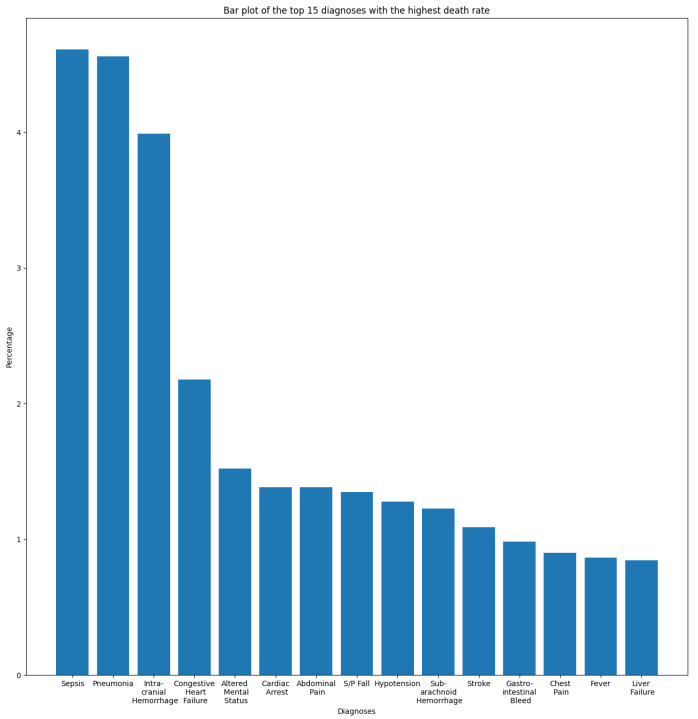
Diagnoses with the highest death rate.

**Figure 7:**
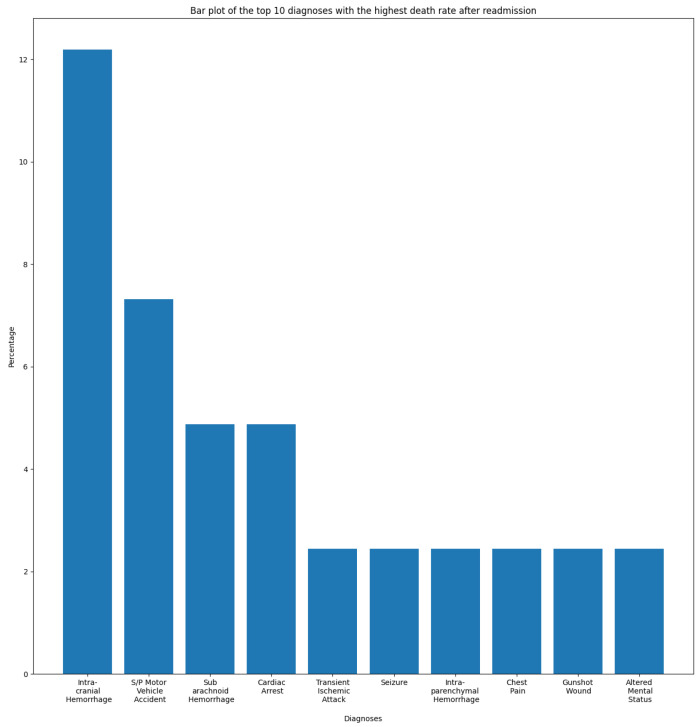
Diagnoses with the highest death rate after readmission.

In this section, we aim to understand and measure the impact of primary diagnoses on readmission rate. Each patient may have multiple diagnoses at the time of admission. A primary diagnosis refers to the one with the highest priority level and which represents the principal cause of admission. To this end, we extracted the most frequent diagnoses from the ADMISSIONS table based on different perspectives. We started our descriptive analysis by extracting the ten most frequent diagnoses for all admissions, which is illustrated in Figure 2. We noticed that Pneumonia was the most frequent diagnosis, with a rate of 2.87%, followed by Sepsis and Coronary Artery Disease, with rates of 2.02% and 1.80%, respectively. The top ten diagnoses represent more than 16% of all 14,221 diagnoses reported in the database. As we are interested in predicting readmission, we extracted the most frequent primary diagnoses of readmitted patients reported before readmission (during their first admission to the hospital) and during readmission, which are illustrated in Figure 3 and Figure 4. The most frequent diagnoses are defined as those diagnoses with the highest number of readmission cases compared to other diagnoses. During our analysis, we first observed that the number of diagnoses reported in readmitted patients does not exceed 1194 diagnoses, compared to 14,221 diagnoses reported in the entire database. We noticed from the figures that 13 diagnoses from these 1194 represented more than 25% of all diagnoses preceding readmission, while only eight diagnoses represented the top 25% of post-readmission diagnoses. At the top of the list are Pneumonia, Congestive Heart Failure, and Sepsis, the three most frequent diagnoses both prior to and after readmission. Other primary diagnoses that were highly present during readmission included Fever, Altered Mental Status, Abdominal Pain, Upper GI Bleed, and Hypotension. From this, it can be deduced that certain diseases probably have a higher impact on the readmission rate compared to others. For further analysis, we used the most frequent diagnoses extracted during the previous analysis to compute the ones with the highest readmission rates. This analysis defines the proportion of readmissions for a diagnosis, computed by dividing the number of readmissions for a diagnosis by the number of all admissions for the same diagnosis. Figure 5 show that Shortness of Breath was the diagnosis with the highest readmission rate, at nearly 14%, followed by Congestive Heart Failure and Abdominal Pain. Other diagnoses with a high readmission rate included Pneumonia, Diabetic Ketoacidosis, and GI Bleed. In addition, we extracted the diagnoses with the highest death rate; Figure 6 and Figure 7 illustrate the bar plots of the diagnoses with the highest death rates before and after readmission, respectively.

Except for Pneumonia, Sepsis, and Congestive Heart Failure, the diagnoses with the highest death rates were somewhat different from the ones with the highest readmission rates. It can be observed from the figures that Intracranial Hemorrhage was the main cause of death after readmission and the third cause for all admission types, while it had a lower readmission rate compared to other diagnoses. In addition, we noticed that the diagnoses with the highest death rates, in particular those after readmission, were not same as those with highest readmission rates. Consequently, from this analysis we assumed that patients suffering from Pneumonia, Congestive Heart Failure, Diabetes, Chest Pain, or GI Bleed present high risk of readmission.

### 2.3. Data Preprocessing

As mentioned above, the ADMISSIONS table contains 58,976 recordings belonging to 46,520 patients. Therefore, a single patient could have multiple admissions stored in different rows. It contains relevant information about each admission, including patient ID, admission type, diagnosis, time of admission, and time of discharge; 71.33% (42,071) of admissions are described as emergencies, 2.3% (1336) as urgent, 13.3% (7863) as newborn, and 13.1% (7706) as elective. We began the preprocessing step by removing newborn and death admissions from the ADMISSIONS table. As we are interested in unplanned readmissions, we kept only the first ’elective’ admission and filtered out the others in order to retain only emergency readmissions. The remaining data contained 45,321 hospital admissions.

The NOTEEVENTS table contains notes from physicians, including discharge summaries, radiology reports, and ECG reports for each admission. We preprocessed the table by selecting only discharge summaries from all the notes in the table, resulting in 59,652 records. We removed duplicated and null discharge summaries from the selected notes and merged the remaining 43,880 notes with the preprocessed ADMISSIONS table. As cited above, a single patient could have several admissions and discharge notes; as we are interested in predicting the readmission occurrence from a single admission, we chose to process only the first admission for each patient, leading to a final dataset of 33,492 records.

**Figure 8:**
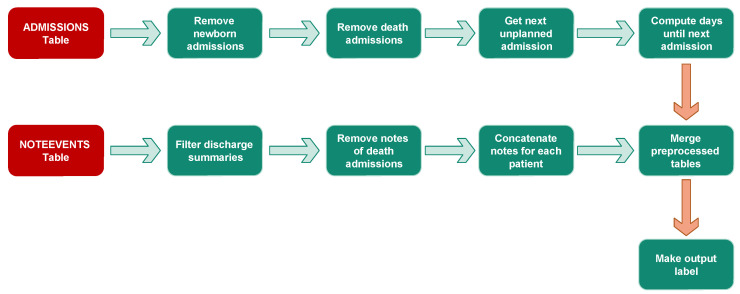
Preprocessing applied to the MIMIC dataset.

**Figure 9:**
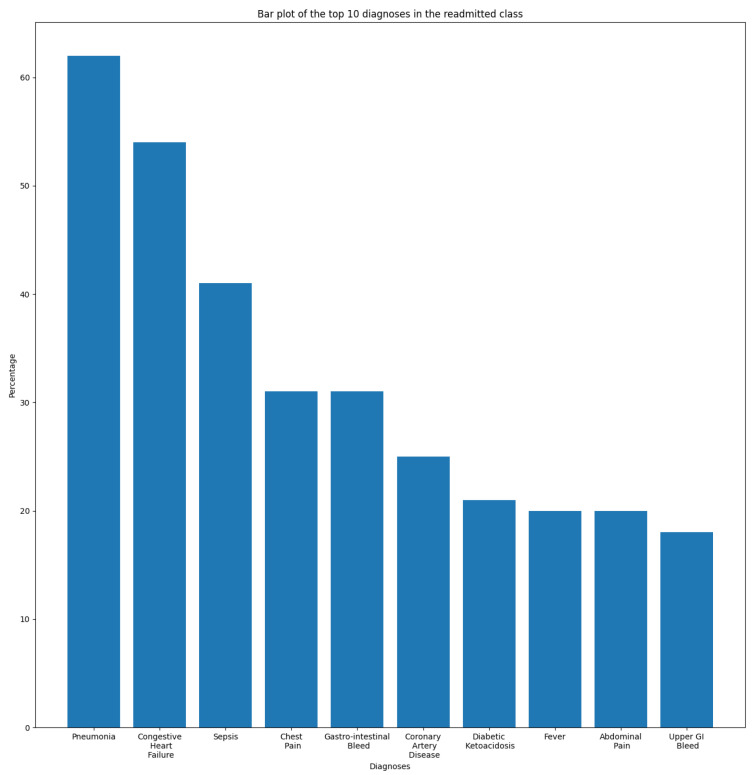
Top ten diagnoses in the “Readmitted” class.

**Figure 10:**
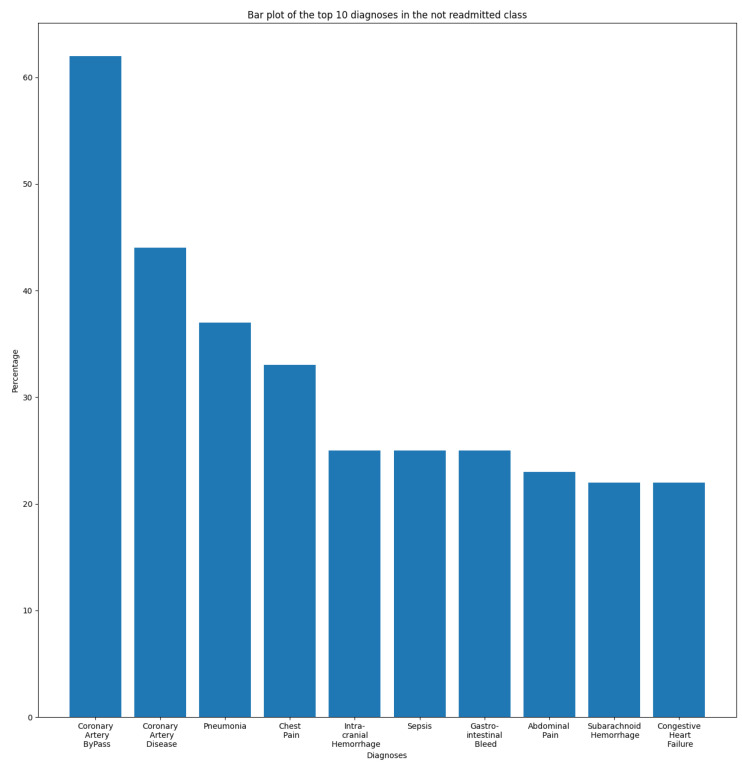
Top ten diagnoses in the “Not readmitted” class.

In this work, we aim to predict whether a patient will experience an unplanned ICU readmission within 30 days of discharge. To this end, we labeled unplanned readmissions that occurred within 30 days of discharge as a positive class, while all others were labeled as negative. The result was an imbalanced dataset with 1900 records for the positive class and 31,592 records for the negative one. Therefore, we subsampled the negative classes by randomly selecting 1900 samples to achieve a 50% prevalence with 3800 samples. Figure 8 illustrates the aforementioned preprocessing steps for both the ADMISSIONS and NOTEEVENTS tables. Figure 9 and Figure 10 illustrate the distribution of the top ten diseases in the balanced dataset for the “readmitted” and “not readmitted” classes, respectively. The two plots support our prior assumption concerning the diseases with the highest impacts on readmission risk. We observed that Pneumonia, Congestive Heart Failure, and Sepsis were the three most frequent diseases among the readmitted class, while Coronary Artery Disease and Coronary Artery Bypass were highly dominant among the negative samples. Accordingly, it can be inferred that the risk of readmission for patients suffering from Coronary Artery Disease or having undergone a Coronary Artery Bypass is relatively low, whereas the risk is significant for those with Pneumonia and Congestive Heart Failure.

After the dataset was created, we preprocessing the clinical notes using NLP techniques before extracting the relevant features for use by the predictive models.

### 2.4. Feature Extraction Using NER

**Figure 11:**
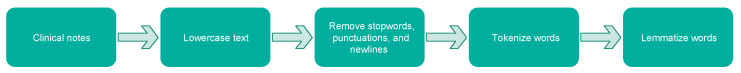
Preprocessing of clinical texts using NLP techniques.

In this section, we aim to extract relevant features for readmission prediction from the discharge notes using NER techniques. Extracting relevant information from clinical notes is a challenging process, as they are essentially unstructured text containing a large amount of information describing patients’ medical records during their admission time, including their age, medications, medical history, diagnosis, laboratory tests, etc. Therefore, a prior preprocessing step is required before handling the named entity recognition task to ensure more accurate results. Figure 11 illustrates the NLP techniques used for the preprocessing step. Among those techniques, we cite the following:

- **Lowercase Text:** The first preprocessing technique was to convert all text to lowercase. NER techniques are case sensitive, with case represented as an important feature for prediction; thus, case uniformity is fundamental in order to avoid biases and treating instances of the same word differently.
- **Stopword Removal:** Removal of stopwords and punctuation is an important process, helping to avoid frequent but unnecessary words that can distract the model from more meaningful words. In addition, this process reduces the dimension of the text and simplifies its representation.
- **Tokenization:** One of the most important preprocessing steps, tokenization breaks a sentence up into separate individual words called tokens, which is essential for analysis of the structure and meaning of the text.
- **Lemmatization:** Lemmatization involves reducing tokenized words to their base or dictionary form. This step helps to consolidate similar words.

Next, we applied NER techniques in order to extract medical entities related to the presence of diseases and symptoms from the preprocessed notes. Sometimes known as entity extraction, NER is a natural language processing technique that aims to extract relevant information, referred to as entities, from unstructured text by identifying and classifying key elements. Entities can include names of people, organizations, locations, dates, numerical values, and other specific types of information. In healthcare, NER aims to recognize medical terms such as diseases, medication, clinical measurements, etc. Existing NER techniques used for clinical notes include the following:

- **En_ner_bc5cdr_md:** A spaCy NER model for processing clinical texts trained on the BC5CDR corpus [12] with an F1-score of 84.28%. The model is intended to recognize elements related to diseases and chemical entities in clinical texts.
- **En_ner_craft_md:** A spaCy NER model trained on the CRAFT corpus [13] with an F1-score of 78.01%. This model recognizes entities related to biomedical ontologies.
- **En_ner_jnlpba_md:** A spaCy NER model trained on the JNLPBA corpus [14] with an F1-score of 72.06%. This model is distinguished by its ability to recognize cell, DNA, and RNA entities.
- **En_ner_bionlp13cg_md:** A spaCy NER model trained on the BIONLP13CG corpus [15] with an F1-score of 77.84%. This model recognize entities related to cancer genetics.
- **BioMedical-NER [16]:** An NER model built on a DistilBERT-based uncased model, which is a refined version of the Bidirectional Encoder Representations from Transformers (BERT) model [17,18]. This model uses the publicly available Maccrobat dataset [19], with an F1-score of 91.89%. BioMedical-NER can recognize 107 biomedical entities, including disease disorders, symptoms, diagnostic procedures, lab values, biological structures, etc.

**Figure 12:**
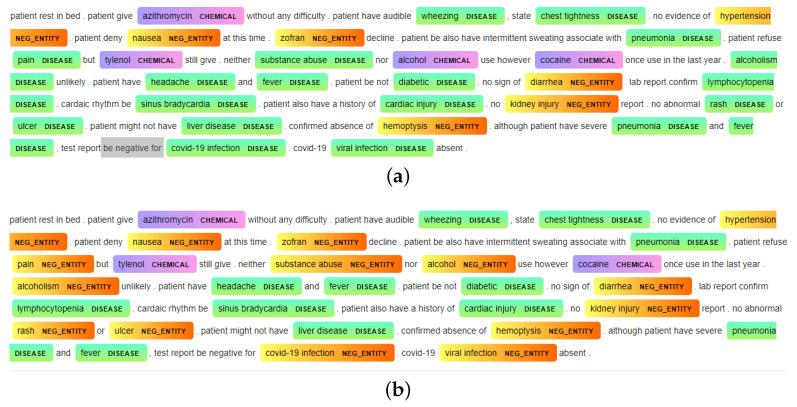
Negation handling using the NegspaCy model: (**a**) before customization and (**b**) after customization.

As we are interested in identifying biomedical entities related to disease and symptoms, BioMedical-NER was the best choice in this case. During the first phase, we fed the preprocessed discharge texts into the pretrained BioMedical-NER model, which returned tokens of the recognized named entities associated with their labels (disease, symptom, clinical events, etc.). However, the clinical notes contained several negative entities that denied the presence of a disease using a negation. We noticed that BioMedical-NER did not consider negative entities when extracting medical entities, and labeled these instances as positive diseases. To tackle this issue, we used the NegspaCy library, which is a spaCy pipeline for negation identification based on the NegEx algorithm [20]. The model detected preceding and following negations such as “deny”, “absence of”, and “no sign of”, successfully labeling the related entities as negative ones; however, it did not consider other negative words as negations, including “no abnormal”, “negative for”, “neither”, “nor”, and “not have”, considering these entities as positive instances. We also noticed that the negation model considered diseases such as renal failure, heart failure, and respiratory failure as negative entities due to the presence of the word failure. To address this concern, we customized the NegspaCy model by including patterns of preceding negation and following negation terms that should be considered by the model. Using the same technique, we removed patterns that contained the term “failure” in order to consider them as positive entities. Figure 12a,b displays the results before and after customizing the NegspaCy model, where each color refers to an entity category (chemical, disease, negative entity). It can be observed that negative entities such as “neither substance abuse”, “nor alcohol”, “no abnormal rash or ulcer”, “negative for COVID-19 infection”, and “COVID-19 viral infection absent” were misidentified as positive words prior to customization.

The extracted entities were then used as input features for the prediction model. However, as this technique generates a boundless amount of features, misleading the prediction model, we created a list of the most important features extracted from the text, represented by the 100 most frequent words related to those diagnoses with the highest readmission rate. This included the names of diseases, symptoms (bleed, pain, fever, vomit, shortness of breath, etc.), and other features that could impact the likelihood of a patient’s early readmission, such as the presence of alcohol or tobacco and the severity level of the illness (mild, severe). As discharge notes may contain acronyms instead of the full name of the disease, our list of features included acronyms for certain diseases, such as CHF for Congestive Heart Failure and chr kidney for chronic kidney.

We then excluded negative entities from the extracted diseases and symptoms, as they indicate the absence of the condition as opposed to its presence.

## 3. Results

The final dataset contained 3800 samples with 99 features, which we split using proportions of 80% for training and 20% for testing. Five ML models were used to train and test the data: K-Nearest Neighbors [21], Decision Tree [22], Random Forest [23], Gradient Boosting [24], and eXtreme Gradient Boosting (XGBoost) [25]. We first trained the dataset using all the features, then applied feature selection techniques to keep only the best predictors. The comparative results are shown in Table 1. The grid search method was used for hyperparameter tuning to adjust the model parameters.

### 3.1. Without Feature Selection

**Figure 13:**
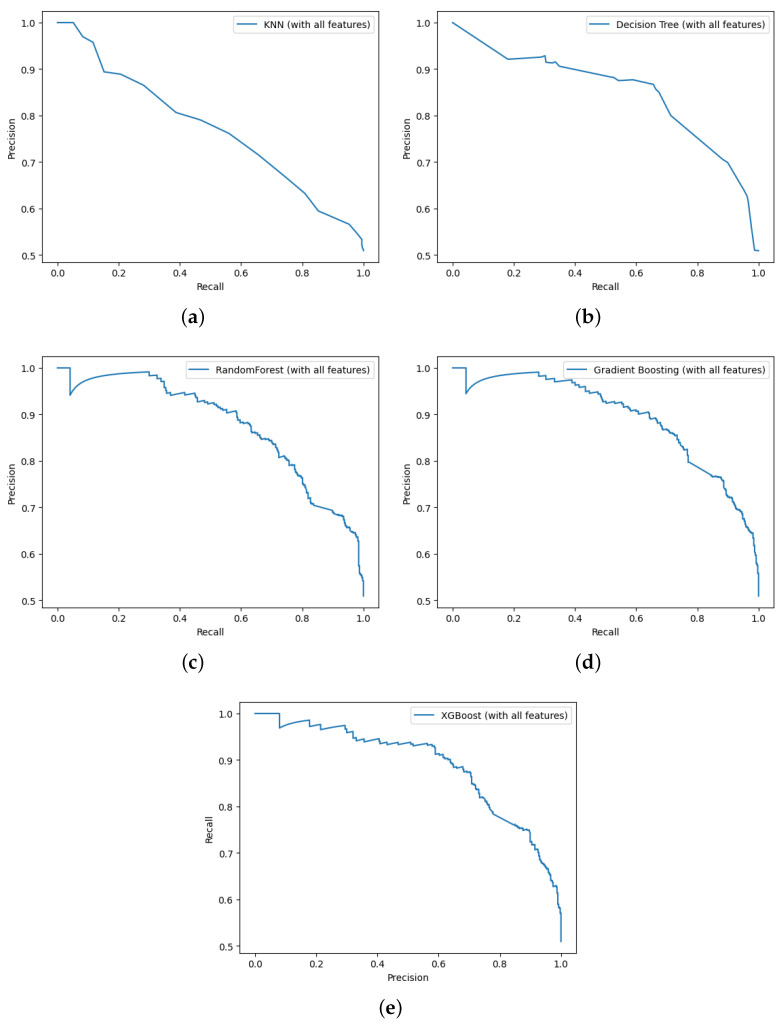
Precision–recall curve of ML models trained on all features: (**a**) KNN, (**b**) Decision Tree, (**c**) Random Forest, (**d**) Gradient Boosting, and (**e**)XGBoost.

**Figure 14:**
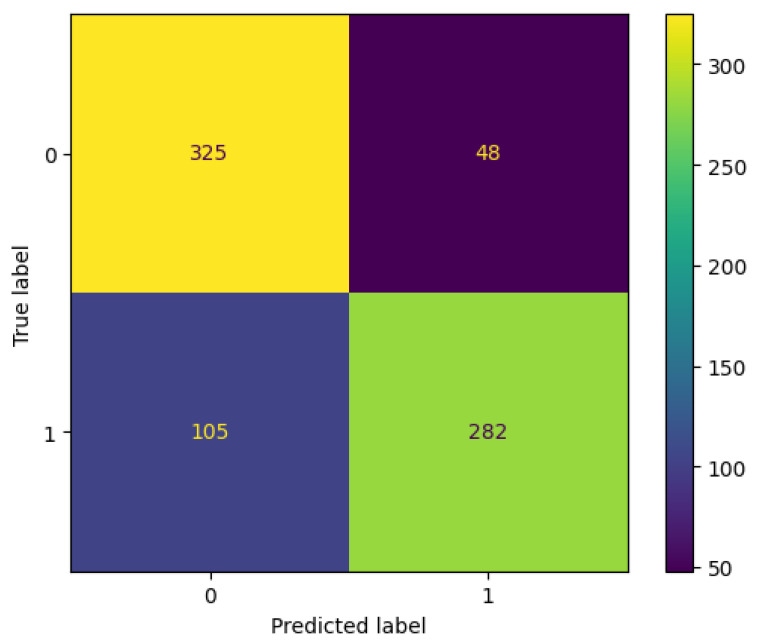
Confusion matrix of the Gradient Boosting model trained on all features.

The models were first trained using the 99 features extracted from the discharge notes. The best performance was achieved by the Gradient Boosting model, with an average precision of 89.4%, followed by XGBoost, with an average precision of 88.8%. Figure 13 illustrates the precision–recall curves for the ML models using all features. The relationship between precision and recall often shows that the precision decreases as the recall increases; thus, a high recall means that the model detects more actual positive cases, which leads to a high rate of false positives and a lower precision. The plot for the Gradient Boosting model is presented in Figure 13d. The plot shows a dip in precision when the recall is close to zero, meaning that although the model made very few positive predictions, it misclassified some of them. However, the precision increases as the model starts to learn from the data, showing a high rate of correct positive predictions. The Gradient Boosting model detects readmission for positive and negative classes with a precision of 85% and 76% and a recall of 73% and 87%, respectively. We can deduce from these results that the GB model can classify readmission with high precision, making only 48 false positive observations out of a total of 332 positive predictions. On the other hand, the number of false negatives is higher for the readmission class, with 105 patients out of a total of 387 misclassified as not readmitted, as illustrated by the confusion matrix in Figure 14.

**Figure 15:**
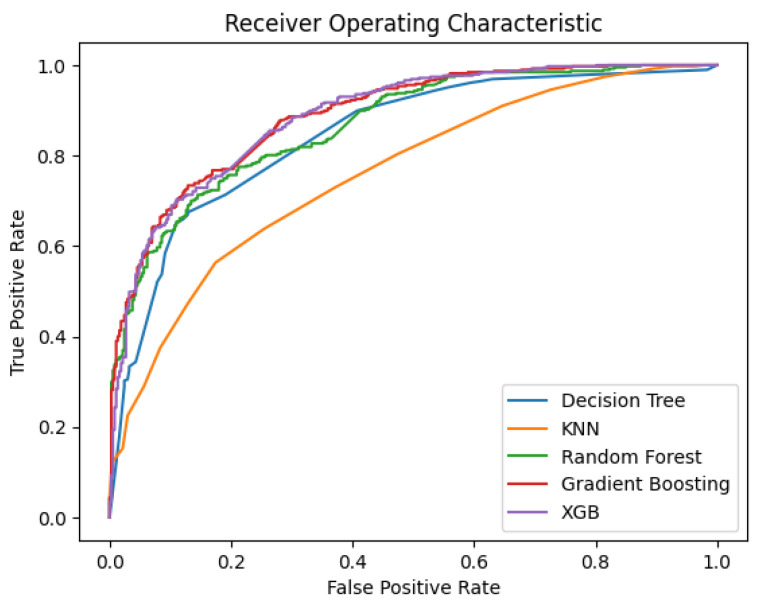
Comparison of the Receiver Operating Characteristic (ROC) curves of the ML models using all features.

Figure 15 illustrates the ROC curves for all features; it can be seen that the Gradient Boosting and XGBoost models have the highest True Positive Rates, with respective AUC-ROC scores of 88.9% and 88.4%.

### 3.2. With Feature Selection

In order to select the best predictors with the highest impact, we applied the *t*-test [26] and decision tree feature selection techniques. Table 2 and Table 3 present the selected features by the *t*-test and decision tree approaches, respectively. It can be seen from the tables that the three best predictors are the same for the both techniques. “Chest” is considered as the feature with the highest impact, with a *p*-value of $1.51\times 10^{-169}$ and an importance of 0.411, followed by “Pain” and “Infarct”. Another important feature identified by both techniques is “Hypertension”. The classification results of the Gradient Boosting and XGBoost models slightly decrease when using the selected features, with the Gradient Boosting model achieving average precision of 87.8% using the 39 features selected by the *t*-test technique and 87.3% using the eight features selected by the decision tree approach and the XGBoost model achieving an average precision of 87.6% and 87.4%, respectively. The performance of the other models increased when using feature selection. The best results were obtained using the decision tree feature selection method, with an average precision of 85.8%, 86.1%, and 86.7% for the KNN, Decision Tree, and Random Forest models, respectively. This difference in performance could be explained by the complexity of the Gradient Boosting and XGBoost models, which are robust at managing high-dimensional data.

The feature selection process aims to select important features using to statistical methods and measure the impact on the classification results of using only those selected features. In our previous work using EHR data [10], models trained on the selected features achieved better performance compared to those trained on all features. However, predicting readmission using only clinical notes is a challenging task, requiring extraction of relevant features from unstructured texts while considering text variability and the fact that relevant features for one text may not necessarily be relevant for another, which could explain the need for a higher number of features.

### 3.3. Cross-Validation

To ensure the generalizability of the models, we applied five-fold cross-validation to the ML models using all features. Table 4, Table 5, Table 6, Table 7 and Table 8 present the cross-validation and training scores with and without feature selection, using accuracy, precision, recall, and F1-score as metrics. The standard deviation was used to measure the score variations for each metric. The results show that for most models the training scores were slightly higher than the cross-validation scores. Further hyperparameter tuning was performed on the Random Forest model to obtain a general model.

**Figure 16:**
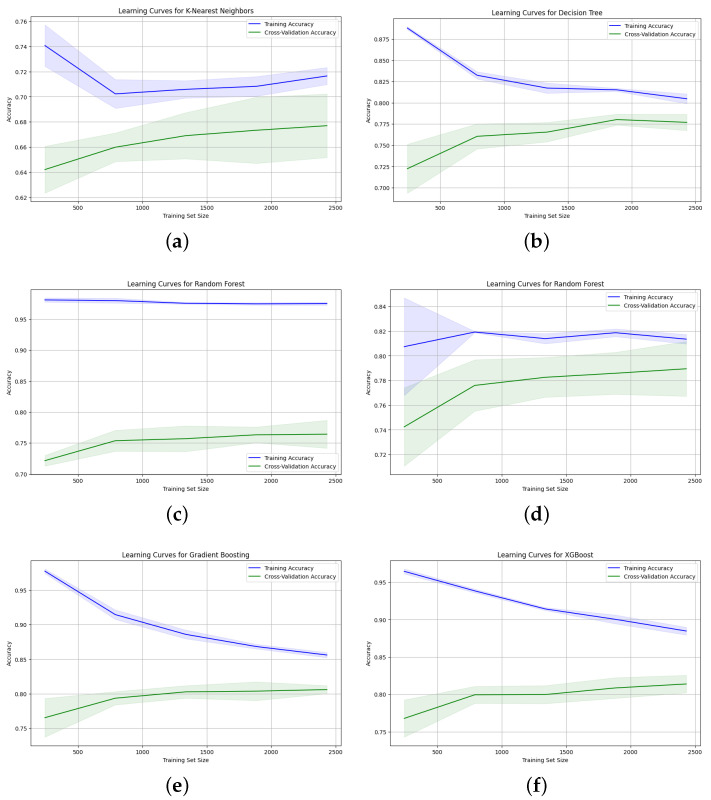
Learning curves for ML models trained on all features: (**a**) KNN, (**b**) Decision Tree, (**c**) Random Forest, (**d**) Random Forest with hyperparameter tuning, (**e**) Gradient Boosting, and (**f**) XGBoost.

For further analysis, we plotted the learning curves for all models, as illustrated in Figure 16. The plots show that the Gradient Boosting (Figure 16e) and Decision Tree (Figure 16b) models were the best at generalizing, as indicated by the convergence of the training and cross-validation accuracy curves. The learning curves for the XGBoost model (Figure 16f) reveal a significant gap between the training and cross-validation curves, with slower improvement in generalization as the dataset size increases. In contrast, the learning curves for the Random Forest model (Figure 16c,d) indicate that the model was overfitting before applying the hyperparameter tuning, and that the model tended to converge when using a configuration of (max_depth = 60, min_samples_leaf = 20, min_samples_split = 50, n_estimators = 500), which significantly decreased the gap between the training and cross-validation accuracy. However, the standard deviation, indicated by the shaded area, is larger in the cross-validation score for the KNN and Random Forest models when using hyperparameter tuning, suggesting high variability in the cross-validation results. Overall, the Gradient Boosting model shows the best performance in terms of both metrics and generalization, achieving high accuracy and precision scores even on unseen data. The XGBoost algorithm also shows consistently high scores on the testing set, but requires a larger dataset to achieve better generalization.

## 4. Explainability

In this section, we use SHapley Additive exPlanations (SHAP) [27] and Local Interpretable Model-agnostic Explanations (LIME) [28] as model-agnostic approaches to aid in understanding and interpreting the prediction results.

### 4.1. SHAP

**Figure 17:**
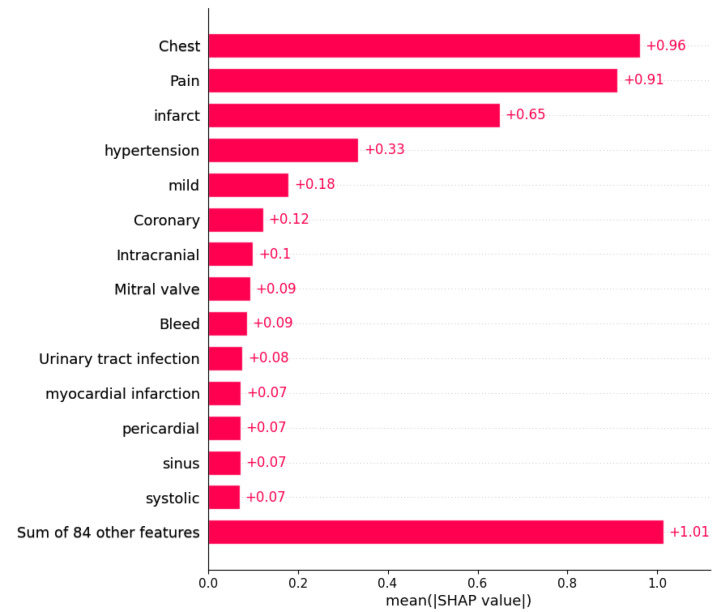
Feature importance using SHAP.

**Figure 18:**
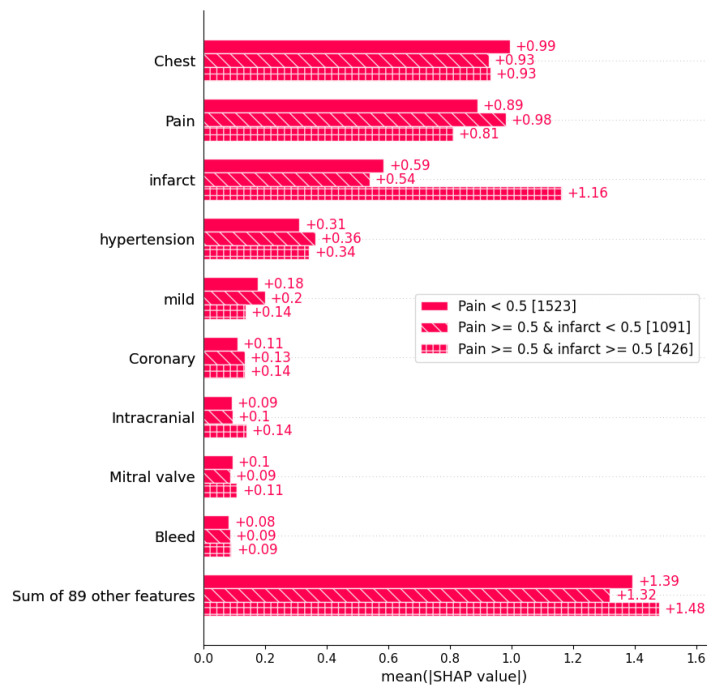
Impact of the “Pain” and “Infarct” features on the prediction.

SHapley Additive exPlanations (SHAP) is an approach used to interpret the features with the highest impact on the decision process. Figure 17 illustrates the most important features considered by the Gradient Boosting model in making predictions. As shown in the figure, the four most important features were exactly the same as those previously selected using the statistical approach based on Student’s *t*-test. However, the rest of the features used by the Gradient Boosting model during the decision process were not previously selected as important features, including “Mild”, “Coronary”, “Intracranial”, “Mitral valve”, and “Bleed”. This may explain the performance decrease of the Gradient Boosting model when using feature selection method. For a more comprehensive analysis, we used a cohort bar plot, shown in Figure 18, to understand the interactions between the most influential features. The figure plots the interaction between the two features with the highest impacts. From the plot, a significant interaction can be observed between the “Pain” and “Infarct” features, with the impact of “Infarct” on the decision process being highly important when both “Pain” and “Infarct” are present (here, ≥0.5 indicates that a feature is present, while <0.5 indicates it is absent). Meanwhile, the importance of the “Chest” feature is higher when “Pain” is absent. It is evident from the plot that the values of “Pain” and “Infarct” play a significant role in the decision process; however, their contributions to the readmission probability remain unclear. Therefore, the LIME approach was used for further analysis.

### 4.2. LIME

**Figure 19:**
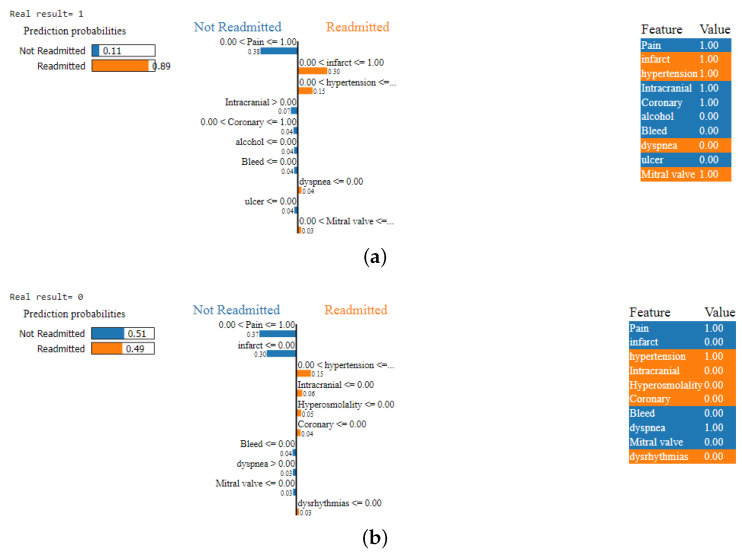
Local explanation for class readmission of correct positive and negative predictions: (**a**) local explanation for class readmission of correct positive prediction and (**b**) local explanation for class readmission of correct negative prediction.

For a deeper analysis, we applied LIME to identify those features that increase the probability of readmission. Figure 19a illustrates the LIME explanations applied to a correctly classified positive readmission sample from the test set. It can be noticed from the figure that the features “Infarct” and “Hypertension” increase the probability of readmission by 45%, while the presence of the “Pain” feature results in more weight being assigned to the “Not Readmitted” class, with a probability of 38%. The absence of other features, including “Alcohol”, “Bleed”, and “Ulcer”, slightly increase the probability of the “Not Readmitted” class by 4% each, which means that their presence increases the readmission probability. Figure 19b presents LIME explanations applied to a correct prediction of the “Not Readmitted" class; the two main features considered in this sample are “Pain” and “Infarct”, where the presence of “Pain” and the absence of “Infarct” increases the probability of the “Not Readmitted” class by 37% and 30%, respectively. However, the presence of “Hypertension” increases the probability of readmission and decreases that of the “Not Readmitted” by 15%. In addition, the presence of “Dyspnea” along with the absence of “Bleed” and “Mitral valve” increases the probability of “Not Readmitted”, for a final prediction probability of 51%.

It can be observe from those two samples that LIME affirms the explainability obtained by SHAP, with “Pain” being the first feature considered by the model during the decision process, followed by “Infarct”, which has more of an impact on the readmission probability when the “Pain” feature is present and consequently decreases the probability of “Not Readmitted” previously increased by “Pain”. Other features, such as “Coronary”, “Intracranial”, “Mitral Valve”, and “Bleed”, are considered important features by SHAP, while LIME provides more clarification about their impact on the prediction probabilities. The presence of “Hypertension”, “Bleed”, and “Mitral Valve” all increase the readmission probabilities, while the absence of these features increases the probability of the “Not Readmitted” class. The presence of “Intracranial”, “Coronary”, and “Dyspnea” all increase the probability of the “Not Readmitted” class, while their absence increases the probability of readmission.

We note that the two explainability methods consider almost the same features with the highest influence on the decision process, with the exception of “Chest”, which is not considered important by LIME. This could be explained as being due the local interpretations of LIME, which only use single predictions to interpret the model, while SHAP provides global explanation.

## 5. Discussion

In this paper, we use discharge notes from the NOTEEVENTS table in the MIMIC-III database to predict the 30-day readmission risk. We use Named Entity Recognition (NER) techniques to extract the most important words related to diseases names and symptoms, which we use as input features for training five different ML models (Gradient Boosting, XGBoost, KNN, Random Forest, and Decision Tree). Feature selection techniques are then used to extract those features (words) with the highest influence on the decision process. The features “Chest”, “Pain”, “Infarct”, and “Hypertension” were selected as the best four predictors, which was later confirmed during the explainability phase of the study. Other features that were selected as significant but with less weight included “Coronary”, “Intracranial”, “Mitral Valve”, “Bleed”, “Infection”, “Congestive Heart Failure”, “Dyspnea”, “Alcohol”, and “Diabetes”.

The presence of the words “Chest”, “Infarct”, “Hypertension”, ”Mitral valve”, or “Congestive Heart Failure” in discharge notes increased the probability of the “Readmitted” class, affirming the output of the descriptive analysis Section 2, where diagnoses related to cardiovascular disease such as “Shortness of Breath”, “Congestive Heart Failure”, and “Chest Pain” were among the most common primary diagnoses of readmitted patients and belonged to the class of diagnoses with the highest readmission rates. The probability of the readmitted class increased evenly with the presence of the “Bleed” feature, which could be related to “Gastrointestinal Bleed (GI Bleed)”, as this was one of the most common primary diagnoses of readmitted patients.

During the explainability phase of this study, we noticed that the presence of the words “Pain”, “Intracranial”, and “Coronary” in a discharge note unexpectedly increased the probability weight of the “Not Readmitted” class. Despite belonging to the top ten primary diagnoses related to ICU admissions, “Coronary” and “Intracranial Hemorrhage” were not considered to be among the primary diagnoses of readmitted patients. Furthermore, patients with an “Intracranial Hemorrhage” diagnosis are subject to a high death risk, as the disease represents the third-leading cause of death for all admission types and the top cause for readmission patients. Therefore, we suppose that the increase in the probability of the “Not Readmitted” class may be due to the the high death rate associated with this diagnosis. However, a physician’s perspective would be more precise in analyzing this case, as well as that of the “Pain” feature. We hypothesize that the features “Chest” and “Pain” are related, and that when both are positive could form a new “Chest Pain” feature. However, this hypothesis was rejected, as when we created the “Chest Pain” feature it was not selected as among the features with the highest impact, while “Chest” and “Pain” individually were both selected.

Our approach achieved better results on the MIMIC-III database compared to other state-of-the-art techniques, with an AUC-ROC of 88.9% compared to 85.8% for BERT and GNN [7], 76.8% for ClinicalBert [8], and 74% for the SVM-RBF model [9]. Predicting readmission using only clinical notes is challenging, as the notes may lack necessary information such as number of hospitalizations, existing comorbidities, and age that is not provided in discharge summaries. To tackle this issue, in future work we will aim to include relevant features from EHR data along with clinical notes for predicting unplanned readmissions.

## 6. Conclusions

In this work, we have aimed to predict 30-day unplanned ICU readmissions using discharge notes from the MIMIC-III database. First, we introduce the topic through a descriptive analysis of diagnoses with high admission, readmission, and death rates. Then, we propose a solution combining NLP and ML techniques. The proposed solution first merges of NER and negation handling techniques to extract features related to positive diseases and symptoms from clinical notes. Next, these features are used as input to several ML models, with the Gradient Boosting model achieving the highest performance at 88.4%. Finally, explainability techniques are applied to assist in understanding the decision-making process of the models and highlight those features with the highest impact on readmission prediction. This work could help to develop a future clinical decision support system for physicians.

## Figures and Tables

**Table 1:** Overview of all models for readmission prediction and comparative results with and without feature selection.

Model	Accuracy	Average Precision (Variance)	F1-Score	Recall	AUC-ROC
**With all features**					
KNN	67%	75.9% (0.037)	59%	47%	76%
Decision Tree	78%	83% (0.021)	75%	67%	85%
Random Forests	78%	87.8% (0.025)	77%	**74%**	86.7%
Gradient Boosting	**80%**	**89.4% (0.025)**	**79%**	73%	**88.9%**
XGBoost	79%	88.8% (0.025)	78%	73%	88.4%
**With student test feature selection (39 features)**					
KNN	75%	83.1% (0.033)	71%	61%	82.8%
Decision Tree	77%	81.3% (0.017)	75%	70%	84.1%
Random Forests	77%	86.8% (0.025)	**77%**	**73%**	85.3%
Gradient Boosting	77%	**87.8% (0.026)**	76%	72%	**86.6%**
XGBoost	**78%**	87.6% (0.024)	76%	72%	86.5%
**Decision Tree feature selection (8 features)**					
KNN	**77%**	85.8% (0.021)	**76%**	**72%**	86.1%
Decision Tree	**77%**	86.1% (0.025)	75%	67%	86.3%
Random Forests	76%	86.7% (0.019)	75%	69%	86.6%
Gradient Boosting	**77%**	87.3% (0.027)	75%	70%	87%
XGBoost	**77%**	**87.4% (0.028)**	75%	70%	**87.1%**
**With statistical feature selection (K = 15)**					
KNN	74%	83.1% (0.026)	73%	68%	83.6%
Decision Tree	**76%**	83.3% (0.020)	74%	67%	84.2%
Random Forests	**76%**	**85.9%(0.020)**	**75%**	**72%**	84.5%
Gradient Boosting	**76%**	85.8% (0.022)	**75%**	71%	**85.1%**
XGBoost	**76%**	85.3% (0.021)	**75%**	70%	84.7%
**With statistical feature selection (K = 10)**					
KNN	74%	83.3% (0.023)	73%	70%	83.1%
Decision Tree	**77%**	83.9% (0.019)	**76%**	70%	84.8%
Random Forests	76%	**85.4% (0.017)**	75%	72%	85.1%
Gradient Boosting	76%	85% (0.018)	**76%**	72%	85.2%
XGBoost	**77%**	85.2% (0.018)	**76%**	**73%**	**85.3%**

**Table 2:** Top 15 features selected as the best predictors using Student’s t-test and Select KBest.

Features	*p*-Value
Chest	$1.51\times 10^{-169}$
Pain	$2.11\times 10^{-121}$
Infarct	$3.42\times 10^{-118}$
Hypertension	$3.53\times 10^{-55}$
Pericardial	$1.25\times 10^{-37}$
Diabete	$4.89\times 10^{-19}$
Pneumothorax	$7.47\times 10^{-18}$
Renal	$4.70\times 10^{-17}$
Infection	$2.88\times 10^{-17}$
Renal Failure	$3.21\times 10^{-16}$
Congestive Heart Failure	$2.75\times 10^{-16}$
Pleural effusion	$1.33\times 10^{-16}$
Insulin	$5.68\times 10^{-14}$
Chronic Kidney	$5.26\times 10^{-14}$
Urinary tract infection	$4.73\times 10^{-14}$

**Table 3:** Features selected as best predictors using the decision tree technique.

Features	Importance
Chest	0.411
Pain	0.287
Infarct	0.141
Myocardial infarction	0.031
Hypertension	0.029
Obstructive Sleep Apnea	0.016
Hematoma	0.013
Coronary	0.011

**Table 4:** Cross-validation results of all models for readmission prediction and comparative results without feature selection.

Model	Accuracy (Std Dev)	Precision (Std Dev)	Recall (Std Dev)	F1-Score (Std Dev)
**Training scores**				
KNN	72.1% (0.013)	86.3% (0.025)	52.2% (0.028)	65% (0.021)
Decision Tree	80.4% (0.003)	85.4% (0.019)	73.3% (0.023)	78.8% (0.006)
Random Forest	81.1% (0.005)	83.7% (0.005)	77.1% (0.008)	80.3% (0.005)
Gradient Boosting	85.5% (0.004)	88.5% (0.003)	81.5% (0.007)	84.8% (0.004)
XGBoost	**88.5% (0.003)**	**91% (0.002)**	**85.2% (0.006)**	**88% (0.003)**
**Cross-validation scores**				
KNN	68.6% (0.017)	82.6% (0.023)	46.7% (0.045)	59.5% (0.034)
Decision Tree	77.6% (0.021)	82.4% (0.011)	70% (0.043)	75.6% (0.028)
Random Forest	78.8% (0.017)	81.1% (0.009)	74.8% (0.031)	77.8% (0.020)
Gradient Boosting	80.7% (0.020)	**83.9% (0.016)**	75.8% (0.029)	79.6% (0.023)
XGBoost	**81.2% (0.015)**	83.3% (0.011)	**77.7% (0.029)**	**80.4% (0.018)**

**Table 5:** Cross-validation results of all models for readmission prediction and comparative results with feature selection based on Student’s t-test (39 features).

Model	Accuracy (Std Dev)	Precision (Std Dev)	Recall (Std Dev)	F1-Score (Std Dev)
**Training scores**				
KNN	77.1% (0.005)	86.4% (0.007)	64% (0.007)	73.5% (0.006)
Decision Tree	80.3% (0.002)	84.4% (0.016)	74.2% (0.023)	78.9% (0.006)
Random Forest	79.8% (0.004)	82.6% (0.009)	75.3% (0.005)	78.8% (0.004)
Gradient Boosting	83.2% (0.002)	86.2% (0.005)	78.9% (0.004)	82.4% (0.002)
XGBoost	**85.5% (0.004)**	**88.5% (0.006)**	**81.4% (0.004)**	**84.8% (0.004)**
**Cross-validation scores**				
KNN	74.2% (0.019)	84.1% (0.012)	59.4% (0.035)	69.6% (0.028)
Decision Tree	77.5% (0.014)	80.9% (0.013)	71.7% (0.033)	76% (0.019)
Random Forest	78.7% (0.016)	81.3% (0.010)	74.2% (0.028)	77.6% (0.019)
Gradient Boosting	**79.3% (0.017)**	**81.9% (0.013)**	**74.9% (0.027)**	**78.2% (0.020)**
XGBoost	79.0% (0.014)	81.5% (0.013)	74.7% (0.023)	77.9% (0.016)

**Table 6:** Cross-validation results of all models for readmission prediction and comparative results using decision tree-based feature selection (eight features).

Model	Accuracy (Std Dev)	Precision (Std Dev)	Recall (Std Dev)	F1-Score (Std Dev)
**Training scores**				
KNN	79% (0.006)	82.3% (0.010)	73.8% (0.009)	77.8% (0.006)
Decision Tree	80% (0.004)	84.5% (0.028)	73.4% (0.032)	78.5% (0.007)
Random Forest	79.2% (0.006)	83.6% (0.016)	72.6% (0.013)	77.7% (0.005)
Gradient Boosting	80.3% (0.004)	84.5% (0.009)	**74.1% (0.009)**	78.9% (0.005)
XGBoost	**80.6% (0.004)**	**85.5% (0.004)**	73.5% (0.006)	**79% (0.005)**
**Cross-validation scores**				
KNN	78.3% (0.013)	81.6% (0.009)	72.9% (0.028)	77% (0.017)
Decision Tree	78.6% (0.016)	83.1% (0.017)	71.5% (0.039)	76.8% (0.022)
Random Forest	78.6% (0.014)	82.6% (0.004)	72.2% (0.036)	77% (0.020)
Gradient Boosting	79.6% (0.014)	83.4% (0.008)	**73.6% (0.027)**	78.2% (0.018)
XGBoost	**80.1% (0.012)**	**84.6% (0.010)**	73.4% (0.027)	**78.6% (0.016)**

**Table 7:** Cross-validation results of all models for readmission prediction and comparative results with SelectKBest-based feature selection (15 features).

Model	Accuracy (Std Dev)	Precision (Std Dev)	Recall (Std Dev)	F1-Score (Std Dev)
**Training scores**				
KNN	77.7% (0.008)	81.7% (0.007)	71.1% (0.013)	76% (0.009)
Decision Tree	79% (0.005)	83.1% (0.012)	72.6% (0.010)	77.5% (0.004)
Random Forest	79% (0.004)	81.7% (0.007)	74.3% (0.004)	77.9% (0.004)
Gradient Boosting	79.9% (0.006)	**82.8% (0.009)**	**75.4% (0.004)**	**78.9% (0.006)**
XGBoost	81.2% (0.005)	84.1% (0.009)	76.6% (0.005)	80.2% (0.005)
**Cross-validation scores**				
KNN	76.1% (0.014)	79.9% (0.013)	69.3% (0.025)	74.2% (0.018)
Decision Tree	77.8% (0.018)	81.6% (0.013)	71.4% (0.032)	76.2% (0.023)
Random Forests	**78.1% (0.014)**	**80.9% (0.007)**	73.2% (0.026)	76.9% (0.017)
Gradient Boosting	**78.1% (0.017)**	80.6% (0.015)	**73.8% (0.023)**	**77% (0.019)**
XGBoost	78% (0.013)	**80.6% (0.012)**	73.5% (0.019)	76.9% (0.015)

**Table 8:** Cross-validation results of all models for readmission prediction and comparative results with SelectKBest-based feature selection (ten features).

Model	Accuracy (Std Dev)	Precision (Std Dev)	Recall (Std Dev)	F1-Score (Std Dev)
**Training scores**				
KNN	77.9% (0.004)	79.8% (0.007)	74.5% (0.006)	77.1% (0.004)
Decision Tree	78.8% (0.004)	82.2% (0.018)	73.5% (0.024)	77.5% (0.007)
Random Forest	78.7% (0.003)	81.6% (0.007)	73.8% (0.006)	77.5% (0.004)
Gradient Boosting	79.2% (0.004)	82.1% (0.009)	74.5% (0.011)	78.1% (0.005)
XGBoost	**80% (0.006)**	**82.9% (0.011)**	**75.5% (0.008)**	**79% (0.005)**
**Cross-validation scores**				
KNN	76.4% (0.017)	78.5% (0.011)	72.5% (0.032)	75.4% (0.021)
Decision Tree	77.2% (0.018)	80.4% (0.015)	71.7% (0.034)	75.8% (0.022)
Random Forest	**78.2% (0.016)**	**81% (0.007)**	73.4% (0.032)	**77% (0.020)**
Gradient Boosting	78.1% (0.017)	80.7% (0.011)	**73.6% (0.030)**	**77% (0.021)**
XGBoost	77.8% (0.013)	80.4% (0.006)	73.2% (0.026)	76.6% (0.017)

## Data Availability

The dataset used in this paper is publicly available (MIMIC-III [11]); see Section 2.1 for more details.
